# Cinchonidia

**Published:** 1877-04-20

**Authors:** 


CINCHONIDIA.—The recent extraordinary advance in the price of the Sulphate of Quinine may make it necessary to fall back on the other preparations of bark. The Cinchonidia has acquired quite a reputation as an antiperiodic in malarial affections. See advertisement of this article by Powers & Weightman, whose establishment has been too long and favorably known to require commendation at our hands.

